# The GC + CC genotype at position -418 in *TIMP-2* promoter and the -1575GA/-1306CC genotype in *MMP-2* is genetic predisposing factors for prevalence of moyamoya disease

**DOI:** 10.1186/s12883-014-0180-5

**Published:** 2014-10-04

**Authors:** Young Seok Park, Young Joo Jeon, Hyun Seok Kim, In Bo Han, Seung-Hun Oh, Dong-Seok Kim, Nam Keun Kim

**Affiliations:** Department of Neurosurgery, Chungbuk National University, College of Medicine, Cheongju-si, South Korea; Institute for Clinical Research, CHA University School of Medicine, Seongnam, South Korea; Department of Neurology, CHA Bundang Medical Center, CHA University School of Medicine, Seongnam, South Korea; Department of Pediatric Neurosurgery, Severance Hospital, Brain Korea 21 Project for Medical Science, Brain Research Institute, Yonsei University College of Medicine, Seoul, Korea

**Keywords:** Moyamoya disease, Tissue inhibitor of metalloproteinase, Matrix metalloproteinases, Polymorphism

## Abstract

**Background:**

To investigate the association of single-nucleotide polymorphisms (SNPs) in matrix metalloproteinases (MMPs)-2, -3, and -9 and tissue inhibitor of metalloproteinase (TIMP)-2 with moyamoya disease (MMD). We conducted a case-control study of MMD patients by assessing the prevalence of six SNPs of *MMP-2* -1575G > A [rs243866]*, MMP-2* -1306C > T [rs243865]*, MMP-3* -1171 5a/6a [rs3025058]*, MMP-9* -1562C > T [rs3918242]*, MMP-9* Q279R [rs17576], and *TIMP-2 -*418G > C [rs8179090].

**Methods:**

Korean patients with MMD (n = 107, mean age, 20.9 ± 15.9 years; 66.4% female) and 243 healthy control subjects (mean age, 23.0 ± 16.1 years; 56.8% female) were included. The subjects were divided into pediatric and adult groups. The genotyping of six well-known SNPs (*MMP-2* -1575G > A*, MMP-2* -1306C > T*, MMP-3* -1171 5a/6a*, MMP-9* -1562C > T*, MMP-9* Q279R, and *TIMP-2 -*418G > C) in *MMP* and *TIMP* genes was performed by polymerase chain reaction-restriction fragment length polymorphism assays.

**Results:**

A significantly higher frequency of the GC genotype for *TIMP-2* -418 G > C was found in MMD patients. The *MMP-9* Q279R GA + AA genotype showed a protective effect for MMD. The GA/CC *MMP-2* -1575/-1306 genotype was significantly more prevalent in MMD patients.

**Conclusions:**

Our findings demonstrate that *TIMP-2* -418 GC + CC and *MMP-2* -1575GA/-1306CC genotypes could be genetic predisposing factors for MMD development.

**Electronic supplementary material:**

The online version of this article (doi:10.1186/s12883-014-0180-5) contains supplementary material, which is available to authorized users.

## Background

The presence of a G/C heterozygous genotype at position -418 in the promoter of the tissue inhibitor of metalloproteinase-2 (*TIMP-2*) gene has been proposed as a genetic predisposing factor for moyamoya disease (MMD) [1], but this association is debated [2]. It is not clear whether there is a genetic effect or an influence of arterial steno-occlusive disease [3]. Although the cause of MMD is still unknown, a genetic background has been strongly suggested, and familial MMD (FMMD) loci have been identified with linkage analyses, supporting a multifactorial inheritance pattern [4-7].

Several studies have demonstrated that overexpression of matrix metalloproteinase-9 (*MMP-9*) and underexpression of *MMP-3, TIMP-1*, and *TIMP-2* are related to MMD [8,9]. Smooth muscle cells (SMC) produce both MMP-2 and-9, and a genetic deficiency in either may decrease SMC invasion and the formation of intimal hyperplasia [10], but no *MMP* genes are located in the loci known to contain *MMD* genes [1]. TIMP dysregulation would disrupt the balance between *MMPs* and *TIMPs* and result in erroneous SMC dynamics, and this could subsequently facilitate MMD development [1]. These findings remain to be confirmed in MMD patients. TIMP dysregulation can disrupt the balance between MMPs and TIMPs, resulting in aberrant SMC dynamics, ultimately leading to MMD [1,2]. Therefore, any single-nucleotide polymorphisms (SNPs) of proteins involved in this cascade may provoke or protect against ischemic or hemorrhagic MMD. Shear stress is very high at the location of proximal internal carotid artery, which might lead to intimal thickening in case of genetic abnormality [11,12]. Dysregulation of *MMPs 2, 3, 9* and their endogenous inhibitor *TIMP-2* was is critical for appropriate extracellular matrix remodeling in response to shear stress in MMD [1,13-15]. MMD can develop in the context of MMP or TIMP genetic susceptibilities and hemodynamic stress. Therefore, we tested whether SNPs of *MMPs 2, 3,* and *9* and *TIMP-2* were associated with MMD in this study.

These genetic abnormalities could facilitate the breakdown of tissue remodeling during moyamoya vessel development, ultimately leading to cerebral ischemia or cerebral hemorrhage. MMD can develop among MMP or TIMP genetic susceptibility against hemodynamic stress.

To test this hypothesis, we conducted a case-control study of MMD patients by assessing the prevalence of six SNPs of *MMP-2, -3, -9* and *TIMP-2* (*MMP-2* -1575G > A [rs243866]*, MMP-2* -1306C > T [rs243865]*, MMP-3* -1171 5a/6a [rs3025058]*, MMP-9* -1562C > T [rs3918242]*, MMP-9* Q279R [rs17576], and *TIMP-2 -*418G > C [rs8179090]).

## Methods

### Subjects

A total of 107 consecutive Korean patients with MMD (mean age, 20.9 ± 15.9 years; 71 females [66.4%], 36 males [33.6%]) were recruited for this study. MMD was defined as the presence of clinical ischemic or hemorrhagic symptoms in combination with vascular lesion evidence on magnetic resonance imaging (MRI) or magnetic resonance angiography (MRA).

The control group was comprised of 243 healthy subjects (mean age, 23.0 ± 16.1 years; 138 female [56.8%]; 105 male [43.2%]) from the same geographic region as the MMD patients. The age- and sex-matched subjects were recruited from outpatient clinics at Severance Hospital (Seoul, Korea) and CHA Bundang Medical Center (Seongnam, Korea). They were healthy volunteers who came in for their regular health examinations. Participants were encouraged to enroll this study, but no incentive as provided to aid recruitment. Control subjects were not related to the participants but were healthy volunteers who came in for their regular health examinations at our university-based hospital.

MMD has a bimodal pattern of incidence, so we divided the patients into pediatric (<18 years) and adult (≥18 years) subgroups. We further divided the MMD patients into ischemic or hemorrhagic subgroups based on clinical and MRI findings. We performed indirect bypass surgery in 64 patients and direct superficial temporal artery to middle cerebral artery bypass plus encephalo-duro-arterio-myo-synangiosis (STA-MCA plus EDAMS) in one patient. Table 1 shows the demographic characteristics of the MMD patients and control subjects.

**Table 1:** **Demographic characteristics between controls and moyamoya patients**

**Characteristic**	**Control (n = 243)**	**Moyamoya (n = 107)**	P*	**Ischemic moyamoya (n = 92)**	**Hemorrhagic moyamoya (n = 15)**	P*
Number of subjects						
<18 years	102 (42.0)	56 (52.3)		54 (58.7)	2 (13.3)	
≥18 years	141 (58.0)	51 (47.7)		38 (41.3)	13 (86.7)	
Age (means ± SD)						
<18 years	7.71 ± 4.05	7.98 ± 4.13	0.92	8.11 ± 4.12	4.50 ± 3.54	NA
≥18 years	36.72 ± 10.05	34.98 ± 11.29	0.25	34.63 ± 11.45	36.00 ± 11.21	0.69
Sex [male, n(%)]						
<18 years	54 (52.9)	22 (39.3)	0.10	21 (38.9)	1 (50.0)	1.00^†^
≥18 years	51 (21.0)	14 (27.5)	0.26	12 (31.6)	2 (15.4)	0.47^†^
Collateral vessel formation score (n = 64)						
0	-	2				
1	-	22				
2	-	40				

*P values were calculated using the Mann-Whitney test for continuous data and χ^2^-test for categorical data.

^†^Fisher’s exact test. NA; not applicable.

All participants provided written informed consent prior to study enrollment. The institutional review boards of Severance Hospital (4-2008-0308) and CHA Bundang Medical Center (PBC09-103,BD 2012-136D,BD 2012-136GR) approved this study.

### Genotyping

DNA was extracted from leukocytes using a G-DEX™ II Genomic DNA Extraction kit (Intron Biotechnology, Seongnam, Korea) according to the manufacturer’s instructions.

For each of the SNPs, 30% of the polymerase chain reaction (PCR) assays were randomly chosen for a second PCR assay followed by DNA sequencing to validate the restriction fragment length polymorphism RFLP findings. Sequencing was performed using an ABI3730xl DNA Analyzer (Applied Biosystems, Foster City, CA, USA). The concordance of the quality control samples was 100%. Each of genotyping methods are described in detail in the Additional file 1.

### Statistical analysis

To analyze the demographic characteristics of MMD, we performed Mann–Whitney U tests and chi-square (χ^2^) tests for continuous and categorical data, respectively. The associations among pediatric and adult patients were estimated by computing the odds ratios (ORs) and 95% confidence intervals (CIs) using Fisher’s exact tests. The adjusted ORs (AORs) for *MMP* and *TIMP* SNPs were calculated using multiple logistic regression analyses using sex and age. Deviations of genotype proportions from Hardy-Weinberg equilibrium (HWE) were tested at each locus, and those of all loci were *p* > 0.01. We marked reference group in tables. The usual type for each locus was chosen as the reference group. Regression coefficient of statistically significant model in detail in the Additional file 2. Statistical analyses were performed using GraphPad Prism 4.0 (GraphPad Software, Inc., San Diego, CA, USA) and StatsDirect software (version 2.4.4; StatsDirect Ltd., Altrincham, UK).

## Results

Table 1 compares the demographic characteristics between controls and MMD patients. The genetic distributions of *MMP-2*, -*3*, and *-9* SNPs are shown in Table 2. Among these, the dominant type (GG vs. GA + AA) of *MMP-9* Q279R (rs17576) was significantly different by χ^2^ test but not by false-positive discovery rate-adjusted *p*-value (Table 2). The genetic distributions of *MMP-2* -1575 G > A, *MMP-2* -1306 C > T, and *MMP-3*-1171 5a/6a were not significantly different between control and MMD. Table 3 shows the genotype frequencies of *MMP* SNPs between the control group and patients with MMD according to age. There was no age-specific differences among the *MMP-2* -1575G > A (rs243866), *MMP-2* -1306C > T (rs243865), *MMP-3* -1171 5a/6a (rs3025058), *MMP-9* -1562C > T (rs3918242), or *MMP-9* Q279R (rs17576) genotypes (Table 3).

**Table 2:** **The genotype frequencies of** ***MMP*** **polymorphisms between the control group and patients with moyamoya disease**

**Characteristic**	**Control (n = 243)**	**Moyamoya (n = 107)**	**AOR (95% CI)**	***P*** ^**a**^	***P*** ^**b**^
*MMP2* -1575G > A (rs243866)					
GG	210 (86.4)	92 (86.0)	1.00 (reference)		
GA	33 (13.6)	15 (14.0)	1.03 (0.53-2.00)	0.94	0.94
AA	0 (0.0)	0 (0.0)	NA	NA	
Dominant (GG vs. GA + AA)			1.03 (0.53-2.00)	0.94	0.94
Recessive (GG + GA vs. AA)			NA	NA	
HWE P	0.256	0.436			
*MMP2* -1306C > T (rs243865)					
CC	222 (91.4)	99 (92.5)	1.00 (reference)		
CT	21 (8.6)	8 (7.5)	0.87 (0.37-2.05)	0.75	0.75
TT	0 (0.0)	0 (0.0)	NA	NA	
Dominant (CC vs. CT + TT)			0.87 (0.37-2.05)	0.75	0.75
Recessive (CC + CT vs. TT)			NA	NA	
HWE P	0.481	0.688			
*MMP3* -1171 5a/6a (rs3025058)					
6a6a	187 (77.0)	78 (72.9)	1.00 (reference)		
6a5a	51 (21.0)	23 (21.5)	1.07 (0.61-1.89)	0.81	0.81
5a5a	5 (2.1)	6 (5.6)	2.92 (0.85-10.00)	0.09	0.18
Dominant (6a6a vs. 6a5a + 5a5a)			1.24 (0.74-2.10)	0.42	0.56
Recessive (6a6a + 6a5a vs. 5a5a)			3.00 (0.88-10.20)	0.08	0.18
HWE P	0.493	0.027			
*MMP9* -1562C > T (rs3918242)					
CC	195 (80.2)	85 (79.4)	1.000 (reference)		
CT	47 (19.3)	19 (17.8)	0.91 (0.50-1.66)	0.76	0.92
TT	1 (0.4)	3 (2.8)	6.12 (0.62-60.42)	0.12	0.24
Dominant (CC vs. CT + TT)			1.03 (0.58-1.83)	0.92	0.92
Recessive (CC + CT vs. TT)			6.45 (0.65-63.68)	0.11	0.24
HWE P	0.298	0.149			
*MMP9* Q279R (rs17576)					
GG	100 (41.2)	56 (52.3)	1.000 (reference)		
GA	120 (49.4)	46 (43.0)	0.66 (0.41-1.07)	0.09	0.11
AA	23 (9.5)	5 (4.7)	0.36 (0.13-1.00)	0.05	0.10
Dominant (GG vs. GA + AA)			0.61 (0.39-0.98)	0.04	0.10
Recessive (GG + GA vs. AA)			0.45 (0.16-1.22)	0.11	0.11
HWE P	0.127	0.244			

Adjusted by age and gender. NA; Not applicable.

^a^ *P* value obtained by Fisher’s exact test.

^b^False positive discovery rate-adjusted *P* value.

**Table 3:** **The genotype frequencies of** ***MMP*** **polymorphisms according to age of participants**

	**Age <18**					**Age ≥18**				
**Characteristic**	**Control (n = 102)**	**Moyamoya (n = 56)**	**AOR (95% CI)**	***P*** ^**a**^	***P*** ^**b**^	**Control (n = 141)**	**Moyamoya (n = 51)**	**AOR (95% CI)**	***P*** ^**a**^	***P*** ^**b**^
*MMP-2* -1575G > A										
GG	88 (86.3)	46 (82.1)	1.00 (reference)			122 (86.5)	46 (90.2)	1.00 (reference)		
GA	14 (13.7)	10 (17.9)	1.32 (0.54-3.23)	0.55	0.55	19 (13.5)	5 (9.8)	0.73 (0.26-2.08)	0.56	0.56
AA	0 (0.0)	0 (0.0)	NA	NA		0 (0.0)	0 (0.0)	NA	NA	
Dominant (GG vs. GA + AA)			1.32 (0.54-3.23)	0.55	0.55			0.73 (0.26-2.08)	0.56	0.56
Recessive (GG + GA vs. AA)			NA	NA				NA	NA	
*MMP-2* -1306C > T										
CC	90 (88.2)	53 (94.6)	1.00 (reference)			132 (93.6)	46 (90.2)	1.00 (reference)		
CT	12 (11.8)	3 (5.4)	0.44 (0.12-1.65)	0.22	0.22	9 (6.4)	5 (9.8)	1.63 (0.51-5.17)	0.41	0.41
TT	0 (0.0)	0 (0.0)	NA	NA		0 (0.0)	0 (0.0)	NA	NA	
Dominant (CC vs. CT + TT)			0.44 (0.12-1.65)	0.22	0.22			1.63 (0.51-5.17)	0.41	0.41
Recessive (CC + CT vs. TT)			NA	NA				NA	NA	
*MMP-3* -1171 5a/6a										
6a6a	76 (74.5)	43 (76.8)	1.000 (reference)			111 (78.7)	35 (68.6)	1.00 (reference)		
6a5a	24 (23.5)	10 (17.9)	0.73 (0.32-1.69)	0.46	0.61	27 (19.1)	13 (25.5)	1.52 (0.71-3.27)	0.28	0.28
5a5a	2 (2.0)	3 (5.4)	3.03 (0.47-19.31)	0.24	0.48	3 (2.1)	3 (5.9)	3.17 (0.61-16.45)	0.17	0.28
Dominant (6a6a vs. 6a5a + 5a5a)			0.90 (0.42-1.95)	0.80	0.80			1.69 (0.82-3.46)	0.15	0.28
Recessive (6a6a + 6a5a vs. 5a5a)			3.55 (0.56-22.60)	0.18	0.48			2.89 (0.56-14.94)	0.21	0.28
*MMP-9* -1562C > T										
CC	79 (77.5)	45 (80.4)	1.00 (reference)			116 (82.3)	40 (78.4)	1.00 (reference)		
CT	22 (21.6)	9 (16.1)	0.62 (0.257-1.493)	0.29	0.39	25 (17.7)	10 (19.6)	1.22 (0.54-2.79)	0.63	0.63
TT	1 (1.0)	2 (3.6)	3.79 (0.33-43.80)	0.29	0.39	0 (0.0)	1 (2.0)	NA	NA	
Dominant (CC vs. CT + TT)			0.75 (0.33-1.71)	0.49	0.49			1.34 (0.60-3.00)	0.47	0.63
Recessive (CC + CT vs. TT)			4.24 (0.37-48.86)	0.25	0.39			NA	NA	
*MMP-9* Q279R										
GG	41 (40.2)	29 (51.8)	1.000 (reference)			59 (41.8)	27 (52.9)	1.00 (reference)		
GA	52 (51.0)	25 (44.6)	0.70 (0.35-1.38)	0.30	0.30	68 (48.2)	21 (41.2)	0.65 (0.33-1.29)	0.22	0.29
AA	9 (8.8)	2 (3.6)	0.25 (0.05-1.29)	0.10	0.30	14 (9.9)	3 (5.9)	0.41 (0.10-1.60)	0.20	0.29
Dominant (GG vs. GA + AA)			0.63 (0.32-1.24)	0.19	0.30			0.62 (0.32-1.18)	0.15	0.29
Recessive (GG + GA vs. AA)			0.38 (0.08-1.83)	0.23	0.30			0.52 (0.14-1.92)	0.33	0.33

Adjusted by age and gender. NA; Not applicable.

^a^ *P* value obtained by Fisher’s exact test.

^b^False positive discovery rate-adjusted *P* value.

In Table 4, the GA/CC-combined genotype of *MMP-2* -1575/-1306 was significantly different in the pediatric group (Table 4). The GC sequence of *TIMP-2* -418 (rs8179090) was significantly different from control (Table 5). The dominant (GG vs. GC + CC) genotype of *TIMP-2* -418 was more frequent in patients with MMD. In the subgroup analysis shown in Table 6, the GC sequence of *TIMP-2* -418 (rs8179090) was significantly different from controls in the adult group. The dominant (GG vs. GC + CC) genotype was more common in adult MMD patients.

**Table 4:** **The combined genotype frequencies of** ***MMP*** **polymorphisms according to age of participants**

	**Age <18**					**Age ≥18**				
**Characteristic**	**Control (n = 102)**	**Moyamoya (n = 56)**	**AOR (95% CI)**	***P*** ^**a**^	***P*** ^**b**^	**Control (n = 141)**	**Moyamoya (n = 51)**	**AOR (95% CI)**	***P*** ^**a**^	***P*** ^**b**^
*MMP-2* -1575/-1306										
GG/CC	88 (86.3)	46 (82.1)	1.00 (reference)			122 (86.5)	46 (90.2)	1.00 (reference)		
GG/CT	0 (0.0)	0 (0.0)	NA	NA		0 (0.0)	0 (0.0)	NA	NA	
GG/TT	0 (0.0)	0 (0.0)	NA	NA		0 (0.0)	0 (0.0)	NA	NA	
GA/CC	2 (2.0)	7 (12.5)	6.70 (1.34-33.60)	0.01	0.02	10 (7.1)	0 (0.0)	0.13 (0.01-2.19)	0.07	0.14
GA/CT	12 (11.8)	3 (5.4)	0.48 (0.13-1.78)	0.39	0.39	9 (6.4)	5 (9.8)	1.47 (0.47-4.63)	0.54	0.54
GA/TT	0 (0.0)	0 (0.0)	NA	NA		0 (0.0)	0 (0.0)	NA	NA	
AA/CC	0 (0.0)	0 (0.0)	NA	NA		0 (0.0)	0 (0.0)	NA	NA	
AA/CT	0 (0.0)	0 (0.0)	NA	NA		0 (0.0)	0 (0.0)	NA	NA	
AA/TT	0 (0.0)	0 (0.0)	NA	NA		0 (0.0)	0 (0.0)	NA	NA	
*MMP-9* -1562/Q279R										
CC/GG	25 (24.8)	22 (39.3)	1.00 (reference)			45 (31.9)	19 (37.3)	1.00 (reference)		
CC/GA	45 (44.1)	21 (37.5)	0.53 (0.24-1.16)	0.11	0.28	57 (40.4)	18 (35.3)	0.72 (0.34-1.55)	0.40	0.62
CC/AA	9 (8.8)	2 (3.6)	0.24 (0.05-1.25)	0.09	0.28	14 (9.9)	3 (5.9)	0.44 (0.11-1.76)	0.25	0.62
CT/GG	15 (14.7)	5 (8.9)	0.50 (0.15-1.71)	0.27	0.44	14 (9.9)	7 (13.7)	1.32 (0.45-3.91)	0.62	0.62
CT/GA	7 (6.9)	4 (7.1)	0.59 (0.14-2.43)	0.46	0.46	11 (7.8)	3 (5.9)	0.66 (0.16-2.66)	0.56	0.62
CT/AA	0 (0.0)	0 (0.0)	NA	NA		0 (0.0)	0 (0.0)	NA	NA	
TT/GG	1 (1.0)	2 (3.6)	3.73 (0.24-58.01)	0.35	0.44	0 (0.0)	1 (2.0)	NA	NA	
TT/GA	0 (0.0)	0 (0.0)	NA	NA		0 (0.0)	0 (0.0)	NA	NA	
TT/AA	0 (0.0)	0 (0.0)	NA	NA		0 (0.0)	0 (0.0)	NA	NA	

Adjusted by age and gender. NA; Not applicable.

^a^ *P* value obtained by Fisher’s exact test.

^b^False positive discovery rate-adjusted *P* value.

**Table 5:** **The genotype frequencies of** ***TIMP-2*** **-418G > C polymorphism between the control group and patients with moyamoya disease**

**Characteristic**	**Control (n = 243)**	**Moyamoya (n = 107)**	**AOR (95% CI)**	***P*** ^**a**^	***P*** ^**b**^
*TIMP-2* -418G > C (rs8179090)					
GG	178 (73.3)	56 (52.3)	1.00 (reference)		
GC	61 (25.1)	46 (43.0)	2.33 (1.42-3.80)	<.01	0.02
CC	4 (1.6)	5 (4.7)	3.53 (0.89-13.98)	0.07	0.09
Dominant (GG vs. GC + CC)			2.39 (1.48-3.85)	<.01	0.02
Recessive (GG + GC vs. CC)			2.32 (0.60-8.96)	0.23	0.23
HWE P	0.64	0.24			

Adjusted by age and gender. NA; Not applicable.

^a^ *P* value obtained by Fisher’s exact test.

^b^False positive discovery rate-adjusted *P* value.

**Table 6:** **The genotype frequencies of** ***TIMP-2*** **-418G > C polymorphism according to age of participants**

	**Age <18**					**Age ≥18**				
**Characteristic**	**Control (n = 102)**	**Moyamoya (n = 56)**	**AOR (95% CI)**	***P*** ^**a**^	***P*** ^**b**^	**Control (n = 141)**	**Moyamoya (n = 51)**	**AOR (95% CI)**	***P*** ^**a**^	***P*** ^**b**^
*TIMP2* -418G > C										
GG	66 (64.7)	29 (51.8)	1.00 (reference)			110 (78.0)	27 (52.9)	1.00 (reference)		
GC	31 (30.4)	23 (41.1)	1.69 (0.84-3.42)	0.14	0.28	31 (22.0)	23 (45.1)	2.99 (1.49-5.98)	<.01	0.01
CC	5 (4.9)	4 (7.1)	1.91 (0.47-7.75)	0.37	0.49	0	1 (2.0)	NA	NA	
Dominant			1.69 (0.86-3.29)	0.13	0.28			3.10 (1.56-6.18)	<.01	0.01
Recessive			1.36 (0.34-5.38)	0.67	0.67			NA	NA	

Adjusted by age and gender. NA; Not applicable.

^a^ *P* value obtained by Fisher’s exact test.

^b^False positive discovery rate-adjusted *P* value.

Genetic impairment of *TIMP-2* and *MMP-2* related with MMD vascular repair gene. We found an abnormality in the GA/CC combined genetic sequence in *MMP-2* -1575/-1306 and the GC sequence of *TIMP-2* -418 (rs8179090), as well as the dominant type (GG vs. GC + CC) in MMD.

## Discussion

In this study, we found that the presence of a G/C heterozygous genotype at position -418 in the *TIMP-2* (rs8179090) promoter, *MMP-2* -1575GA/-1306CC, and the dominant type (GG vs. GA + AA) of *MMP-9* Q279R (rs17576) could be genetic predisposing factors for MMD. By degrading the neurovascular matrix, MMPs promote blood-brain barrier (BBB) damage, edema, and hemorrhage [13,16,17]. Several studies have demonstrated that overexpression of MMP-9 and underexpression of MMP-3, TIMP-1, and TIMP-2 are related to MMD [8,9].

The balance between MMPs and TIMPs is known to be an important factor of BBB maintenance and vascular angiogenesis [18]. MMP-2 and -9 are able to digest the endothelial basal lamina, which plays a major role in maintaining BBB impermeability by regulating tight junctions leading to the opening of BBB [19]. MMP-2 and MMP-9 released from the vascular endothelium and leukocytes during the inflammatory phase of ischemic stroke use collagen IV and laminin as substrates [20,21]. Serum MMP-9 levels were significantly higher in patients with MMD compared to that in healthy controls [8,9]. It is conceivable that MMP-9 upregulation may contribute, at least in part, to the breakdown of BBB structure, including endothelial basal lamina, and thereby facilitate hemorrhage development [9,22]. Any genetic abnormality or hemodynamic stress raises the possibility of BBB breakdown in patients with predisposing MMP or TIMP gene susceptibility. MMD can develop among MMP or TIMP genetic susceptibility against hemodynamic stress.

Several SNPs in the promoters of *MMP* genes have been demonstrated to affect the expression levels of corresponding proteins [23-26]. Allelic effects on transcriptional activity have also been demonstrated for *MMP-2* C–735 T, *MMP-3* –1171 5a/6a, and *MMP-13* G–77A SNPs [25,27,28]. *MMP-3* can degrade a number of ECM proteins and activate several other MMPs, the 6a allelic variant identified at position –1171 in the *MMP-3* promoter exhibits lower promoter and transcriptional activity than the 5a allele [25], and homozygosity of the 6a allele was associated with common carotid geometry and carotid artery atherosclerosis [29,30].

Here, we investigated five SNPs in MMPs and one SNP in TIMP. Previous studies have reported associations between MMD and expression levels of MMPs and TIMPs [8,9]. TIMPs are the most important endogenous inhibitors of MMPs, in particular TIMP-1 and TIMP-2. Therefore, SNPs that lead to structural defects or modify the transcription rate of TIMP-2 could affect BBB breakdown and thereby influence the magnitude and/or incidence of ischemic stroke and intracranial hemorrhage [31]. SNPs can also interfere with the balance of MMPs and TIMP-2 in the absence of acute BBB disruption, thereby influencing the development and severity of atherosclerosis, white matter lesions, and small-vessel disease [31].

While TIMP-2 has already been demonstrated to play a role in MMD, it is important to replicate and support previous studies. Our results corroborate previous FMMD studies by Kang et al [1], but are different from those reported by other groups [14,15]. The discrepancy might be due to different genetic backgrounds among patient populations.

The major strength of this study is that we were able to replicate previous findings by performing a case-control study with a relatively large number of MMD patients. Our findings provide additional evidence that the G/C genotype -418 of *TIMP-2* is more prevalent in individuals with MMD.

Potential weaknesses of this study are that the sample did not include patients with familial MMD, and family pedigrees were not assessed. Also, as this was an association study with a case-control study design, independent cohort studies are needed to confirm our findings. We did not perform a correlation study with blood MMP and TIMP levels. We selected only a few *MMP* and *TIMP* candidate SNPs; therefore, more genetic sequences would be needed to reach stronger conclusions. In addition, the small sample size may have resulted in a Type I error. The inconsistency between the family- and population-based studies could be due to various reasons, and more compelling evidence is needed to clarify this.

## Conclusions

Our findings demonstrate that the G/C heterozygous genotype in the *TIMP-2-*418G>C (rs8179090) promoter, *MMP-2* -1575GA/-1306CC, and the dominant type (GG vs. GA + AA) of *MMP-9* Q279R (rs17576) could be genetic predisposing factors for MMD development. These genetic polymorphisms can lead to the breakdown of tissue remodeling during MMD progression, which could lead to cerebral ischemia or cerebral hemorrhage. These results are consistent with previous studies of the genetic dysregulation of vascular repair mechanisms.

## Additional files

Additional file 1
**Genotyping.**
Additional file 2: Table S1 Regression coefficient of statistically significant models.
