# Supplementary material for: The GC + CC genotype at position -418 in TIMP-2 promoter and the -1575GA/-1306CC genotype in MMP-2 is genetic predisposing factors for prevalence of moyamoya disease
Source: BMC Neurol. 2014 Oct 4;14:180. doi: 10.1186/s12883-014-0180-5 (PMC4196131; doi:10.1186/s12883-014-0180-5)
Supplement: Additional file 1 — Genotyping. [file 12883_2014_180_MOESM1_ESM.doc]

**Genotyping**

The *MMP-2* -1575G>A polymorphism was detected by polymerase chain reaction-restriction fragment length polymorphism (PCR-RFLP) analysis using a sense primer (5'– GTC TGA AGC CCA CTG AGA CC –3') and a reverse primer (5'– CTA GGA AGG GGG CAG ATA GG –3'). The 175bp PCR product was then digested with 3U *Nla*III for 16 h at 37°C. A digestion product of 175bp represented the GG genotype; fragments of 175bp, 113bp, and 62bp represented the GA genotype; and fragments of 113bp and 62bp represented the AA genotype.

The *MMP-2* -1306C>T polymorphism was detected by PCR-RFLP analysis. The following primers were used to amplify the *MMP-2* -1306C>T region: forward, 5'– CTT CCT AGG CTG GTC CTT ACT GA –3' and reverse, 5'– CTG AGA CCT GAA GAG CTA AAG AGC T –3'. The 193bp product was digested with 3U *Bfa*I for 16 h at 37°C. A restriction fragment of 193bp represented the CC genotype; fragments of 193bp, 167bp, and 26bp represented the CT genotype; and fragments of 167bp and 26bp represented the TT genotype.

To detect the *MMP-3* -1171 5a/6a genotype, PCR-RFLP analysis was performed with a sense primer (5'– GTT CTC CAT TCC TTT GAT GGG GGG AAA GA –3') and an antisense primer (5'– TTC CTG GAA TTC ACA TCA CTG CCA CCA CT –3'). The length of the amplified fragment was 128bp. PCR products were digested with 3U *pflF*I for 16 h at 37°C. The 128bp product identified the 6a6a genotype; products of 128bp, 95bp, and 34bp represented the 6a5a genotype; and products of 95bp and 34bp represented the 5a5a genotype.

To detect the *MMP-9* -1562C>T genotype, PCR-RFLP analysis was performed with a sense primer (5'– GCC TGG CAC ATA GTA GGC CC –3') and an antisense primer (5'– GGG TTC AAG CAA TTC TCC TG –3'). The length of the amplified fragment was 246bp. PCR products were digested with 3U *Sph*I for 16 h at 37°C. The 246bp product identified the CC genotype; products of 246bp, 194bp, and 52bp represented the CT genotype; and products of 194bp and 52bp represented the TT genotype.

To detect the *MMP-9* Q279R genotype, PCR-RFLP analysis was performed with a sense primer (5'– ACC ATC CAT GGG TCA AAG AA –3') and an antisense primer (5'– GAT TGG CCT TGG AAG ATG AA –3'). The length of the amplified fragment was 161bp. PCR products were digested with 3U *Sma*I for 16 h at 25°C. Restriction products of 103bp and 58bp identified the GG genotype; products of 161bp, 103bp, and 58bp represented the GA genotype; and the 161bp product represented the AA genotype.

The *TIMP-2* -418G>C polymorphism was detected by PCR-RFLP analysis using a sense primer (5'– GGG ATC CTG TCA GTT TCT CAA –3') and a reverse primer (5'– TAC CCT TTC CCC TTC AGC TC –3'). The length of the amplified fragment was 182bp. PCR products were digested with 3U *BsoB*I for 16 h at 37°C. The restriction patterns were as follows: 182bp (GG); 182bp, 98bp, and 84bp (GC); and 98bp and 84bp (CC).
