# Supplementary material for: The GC + CC genotype at position -418 in TIMP-2 promoter and the -1575GA/-1306CC genotype in MMP-2 is genetic predisposing factors for prevalence of moyamoya disease
Source: BMC Neurol. 2014 Oct 4;14:180. doi: 10.1186/s12883-014-0180-5 (PMC4196131; doi:10.1186/s12883-014-0180-5)
Supplement: Additional file 2: Table S1 — Regression coefficient of statistically significant models. [file 12883_2014_180_MOESM2_ESM.doc]

| Supplementary Table 1. Regression coefficient of statistically significant models | | | | |
| --- | --- | --- | --- | --- |
| Association | Group | Coefficient of genotype | Coefficient of age | Coefficient of gender (female) |
| *MMP-9* Q279R GA+AA | Total | -0.494 | -0.033 | 0.447 |
| *MMP-2* -1575GA/-1306CC | Age<18 | 1.902 | 0.023 | 0.319 |
| *TIMP-2* -418 GC+CC | Total | 0.871 | -0.031 | 0.445 |
| *TIMP-2* -418 GC+CC | Age≥18 | 1.131 | -0.053 | 0.222 |
